# Identification of differentially expressed genes of brown trout (*Salmo trutta*) and rainbow trout (*Oncorhynchus mykiss*) in response to *Tetracapsuloides bryosalmonae* (Myxozoa)

**DOI:** 10.1007/s00436-014-4258-1

**Published:** 2015-01-07

**Authors:** Gokhlesh Kumar, Ahmed Abd-Elfattah, Mansour El-Matbouli

**Affiliations:** Clinical Division of Fish Medicine, Department for Farm Animals and Veterinary Public Health, University of Veterinary Medicine, Veterinärplatz 1, 1210 Vienna, Austria

**Keywords:** Salmonids, Myxozoan parasite, Proliferative kidney disease, Transcriptome, Differentially expressed genes

## Abstract

*Tetracapsuloides bryosalmonae* Canning et al., 1999 (Myxozoa) is the causative agent of proliferative kidney disease in various species of salmonids in Europe and North America. We have shown previously that the development and distribution of the European strain of *T. bryosalmonae* differs in the kidney of brown trout (*Salmo trutta*) Linnaeus, 1758 and rainbow trout (*Oncorhynchus mykiss*) Walbaum, 1792, and that intra-luminal sporogonic stages were found in brown trout but not in rainbow trout. We have now compared transcriptomes from kidneys of brown trout and rainbow trout infected with *T. bryosalmonae* using suppressive subtractive hybridization (SSH). The differentially expressed transcripts produced by SSH were cloned, transformed, and tested by colony PCR. Differential expression screening of PCR products was validated using dot blot, and positive clones having different signal intensities were sequenced. Differential screening and a subsequent NCBI-BLAST analysis of expressed sequence tags revealed nine clones expressed differently between both fish species. These differentially expressed genes were validated by quantitative real-time PCR of kidney samples from both fish species at different time points of infection. Expression of anti-inflammatory (TSC22 domain family protein 3) and cell proliferation (Prothymin alpha) genes were upregulated significantly in brown trout but downregulated in rainbow trout. The expression of humoral immune response (immunoglobulin mu) and endocytic pathway (Ras-related protein Rab-11b) genes were significantly upregulated in rainbow trout but downregulated in brown trout. This study suggests that differential expression of host anti-inflammatory, humoral immune and endocytic pathway responses, cell proliferation, and cell growth processes do not inhibit the development of intra-luminal sporogonic stages of the European strain of *T. bryosalmonae* in brown trout but may suppress it in rainbow trout.

## Introduction

Proliferative kidney disease (PKD) significantly affects both farmed and wild salmonid fish in Europe and North America, causes economic losses, and endangers wild fish populations (El-Matbouli and Hoffmann 2002; Okamura et al. 2011). PKD is caused by the myxozoan parasite *Tetracapsuloides bryosalmonae* Canning et al.,1999. Spores develop in the kidney tubules of infected fish and are released via urine to infect freshwater bryozoans (Morris and Adams 2006, 2007; Grabner and El-Matbouli 2008). Overtly infected bryozoans release the *T. bryosalmonae* spores into the water, and the spores enter into the fish host through gills and then migrate to the kidney (Morris et al. 2000; Grabner and El-Matbouli 2010). Proliferation of *T. bryosalmonae* induces a granulomatous cellular response in the interstitial tissue, which leads to swelling of the spleen and kidney (Ferguson and Needham 1978; Clifton-Hadley et al. 1987).

In Europe, mature parasite spores form in the kidney tubules of brown trout (*Salmo trutta*) Linnaeus, 1758 but not in rainbow trout (*Oncorhynchus mykiss*) Walbaum, 1792 (Bucke et al. 1991). However, in North America, rainbow trout can form spores in kidney tubules (Kent and Hedrick 1986; Hedrick et al. 2004). This led to the hypothesis that there are two lineages of *T. bryosalmonae*: one adapted to the genus *Salmo* and the other to the genus *Oncorhynchus* (Bucke et al. 1991; Morris et al. 1997). Internal transcribed spacer sequence data supported this hypothesis by resolving distinct European and North American lineages of *T. bryosalmonae* (Henderson and Okamura 2004). Furthermore, Grabner and El-Matbouli (2008) and Kumar et al. (2013) showed that rainbow trout infected with European strain of *T. bryosalmonae* could not transmit the parasite to bryozoan *Fredericella sultana* Blumenbach, 1779, but infected brown trout could. Recently, we showed that development and distribution of *T. bryosalmonae* in brown trout and rainbow trout vary at different stages of infection and that intra-luminal sporogonic stages of the parasite are present in brown trout but not in rainbow trout (Kumar et al. 2013). Additionally, we verified the persistence of *T. bryosalmonae* in chronically infected brown trout and their ability to infect the bryozoan colonies up to 104 weeks post-exposure (wpe) (Abd-Elfattah et al. 2014).

Suppression subtractive hybridization (SSH) can detect transcripts that are differentially expressed in two RNA samples (Hillmann et al. 2009). SSH has been used successfully for a genetic study on activated and inactivated spores of a different myxozoan, *Myxobolus cerebralis* (Eszterbauer et al. 2009). SSH showed differential expression of immune relevant genes in resistant and susceptible strains of Atlantic salmon (*Salmo salar*) infected with the monogenean, *Gyrodactylus salaris* (Matejusová et al. 2006). SSH has been used to identify differentially expressed genes in the head kidney and intestine of susceptible and resistance gilthead sea bream (*Sparus aurata*) to myxosporean, *Enteromyxum leei* infection (Davey et al. 2011).

We compared transcriptomes from kidneys of brown trout and rainbow trout infected with the European strain of *T. bryosalmonae*. We used SSH to identify transcripts differentially expressed in kidneys of the two fish species. Differential expression of genes was confirmed using quantitative real-time PCR (qRT-PCR) to evaluate relative gene expression levels in the kidneys of both infected brown trout and rainbow trout at different time points.

## Materials and methods

### Ethics statement

This study was approved by the institutional ethics committee of the University of Veterinary Medicine Vienna and the national authority according to §26 of the Austrian Law for Animal Experiments, Tierversuchsgesetz 2012–TVG 2012 91 under the no. GZ 68.205/0247-II/3b/2011.

### Experimental design and fish sampling 

**Fig. 1:**
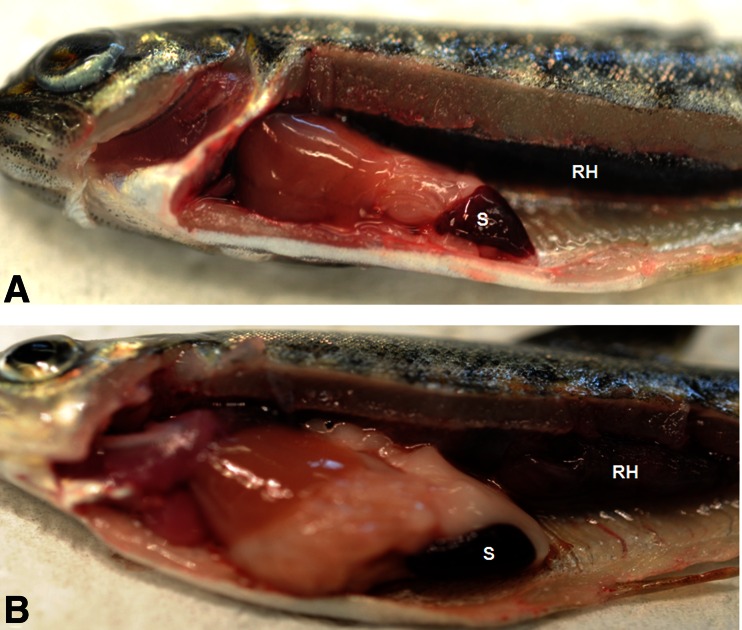
*T* *etracapsuloides bryosalmonae* infecting fish. Brown trout (**a**) and rainbow trout (**b**) showing clinical signs of proliferative kidney disease: renal hypertrophy (*RH*) and splenomegaly (*S*)

Infected fish originated from our previous experiment (Kumar et al. 2013). Briefly, specific pathogen-free 60 brown trout and 60 rainbow trout (mean length 5.5 ± 0.5 cm, mean weight 2.3 ± 0.5 g) were placed in flow-through aquaria. Prior to infection, fish were transferred to a small aquarium and the volume of water in the aquarium was reduced. Free *T. bryosalmonae* spores in suspension released from 12 mature sacs of parasite (single mature sac contains 2800–4000 spores as described by Okamura et al. (2011)) from the laboratory infected *F. sultana* colonies were added to all aquaria which were then maintained with vigorous aeration for 24 h at 16.5 ± 1 °C. After infection, fish were transferred to 100-l volume of water in an aquarium and maintained at 16.5 ± 1 °C. Additional 30 brown trout and 30 rainbow trout were held as non-infected controls. Posterior kidneys were sampled from both infected (*n* = 10) and control (*n* = 5) groups at 6, 8, 10, 12, 14, and 17 wpe. The clinical signs of PKD in both fish species are shown in Fig. 1.

### RNA and mRNA preparation

**Fig. 2:**
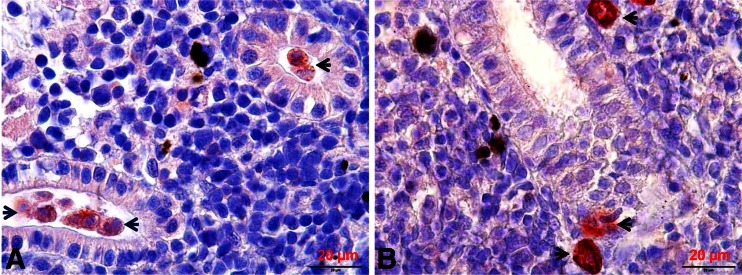
*Tetracapsuloides bryosalmonae* stages in kidney of infected brown trout and rainbow trout. **a** Numerous intra-luminal sporogonic parasite stages (*arrows*) in the kidney of brown trout. **b** Numerous interstitial presporogonic parasite stages (*arrows*) in the kidney of rainbow trout. Parasite stages were visualized by immunohistochemistry using anti-*T. bryosalmonae* monoclonal antibody and counterstained with hematoxylin

The optimal time point for the SSH assay was determined by presence of numerous *T. bryosalmonae* stages, observed using immunohistological examination (Kumar et al. 2013). Briefly, sporogonic stages of *T. bryosalmonae* were observed in the kidney lumen of brown trout but not in the rainbow trout (Kumar et al. 2013). Total RNA was extracted from eight kidneys of each fish species at 8 and 10 wpe, coincident with observation of both high numbers of sporogonic stages (Fig. 2a) and low presporogonic stages in brown trout and only high numbers of presporogonic stages in rainbow trout (Fig. 2b) using a RNeasy mini kit (Qiagen). An on-column DNase digestion step was included to remove any residual DNA contamination (Qiagen). Equal amounts of RNA were pooled to even out differences in RNA between individual fish. Messenger RNA was purified from the pooled RNA sample using an Oligotex mRNA kit (Qiagen).

### Suppression subtractive hybridization 

Two micrograms of mRNA was reverse-transcribed into first-strand complementary DNA (cDNA) using SMARTScribe reverse transcriptase, then into second-strand cDNA by a second-strand enzyme cocktail (Clontech). PCR, using beta-actin primers, was used to access the relative amount of cDNA products after double-strand (ds) cDNA synthesis (Rucker and El-Matbouli 2007).

cDNA pools of infected brown trout and infected rainbow trout were hybridized in two steps. A PCR-Select cDNA subtraction kit (Clontech) was used for SSH. The forward, reverse, and control hybridizations were performed to create subtracted cDNA per manufacturer’s suggestions. For the forward subtraction, infected brown trout cDNA was used as “tester” and infected rainbow trout cDNA as “driver.” For the reverse subtraction, infected rainbow trout cDNA was used as tester and infected brown trout cDNA as driver. The ds cDNA was digested by RsaI restriction enzyme and purified. Ligation and hybridization were performed following the manufacturer’s protocols. The differentially expressed transcripts produced by SSH were cloned into a pCR4 TOPO TA cloning vector (Invitrogen) and transformed into *Escherichia coli* TOP10 cells. All clones were picked and tested by colony PCR using adaptor-specific nested PCR primer 1 (5′-TCGAGCGGCCGCCCGGGCAGGT-3′) and nested PCR primer 2R (5′-AGCGTGGTCGCGGCCGAGGT-3′) in a PCR-Select cDNA subtraction kit (Clontech). PCR reaction was carried out in a final volume of 25 μl containing 12.5 μl of 2× ReddyMix PCR Master Mix (Thermo Scientific), 10 pmol of each primer, sterile distilled water, and individual transformants. PCR amplification was performed as follows: 30 cycles of 94 °C for 45 s, 66 °C for 45 s, and 72 °C for 90 s.

### Differential expression screening 

Differential expression screening of PCR products amplified from clones was performed to validate and explore patterns of gene expression using dot blots. For this experiment, the DIG-High Prime DNA Labeling and Detection Starter Kit I (Roche Applied Science) was used. The cDNA libraries of subtracted forward, subtracted reverse, unsubtracted tester control, and unsubtracted driver control were digested with RsaI and labeled with DIG-digoxigenin, and used as probes for the dot blot hybridization. Analysis of the DIG-labeling efficiency was performed as per the manufacturer’s instructions. To denature the DNA, a final concentration of 0.4 M NaOH/10 mM EDTA was added to each of the PCR products amplified by the adaptor-specific PCR primers (1 and 2R), then placed in a Thermomixer comfort (Eppendorf) at 99 °C for 10 min, then placed on ice. Two microliters of each denatured PCR product was dotted onto a positively charged nylon membrane (Roche Applied Science) and UV cross-linked to the membranes for 3 min. Membranes were prehybridized in DIG Easy Hyb at 42 °C for 30 min and hybridized overnight at 42 °C with DIG-labeled cDNA probes. Hybridization and subsequent detection was performed using anti-DIG-alkaline-phosphatase conjugate and BCIP/NBT substrate following the manufacturer’s protocol. Differential expression of enriched subtracted forward and reverse cDNA libraries were analyzed and compared with unsubtracted cDNA libraries controls.

### Identification of clones and sequencing analysis

Positive clones of subtracted forward and reverse cDNA libraries were selected as having different signal intensities in the dot blot screening, then sequenced at LGC Genomics GmbH, Berlin, Germany. Vector and adaptor-sequences were removed from the expressed sequence tags (ESTs). BLAST searches were performed to identify the ESTs in the non-redundant sequences database using BLASTn and BLASTx tools at the National Center for Biotechnology Information (NCBI).

### Quantitative real-time PCR

From the kidney samples collected at 6, 8, 10, and 12 wpe, total RNA was extracted using the RNeasy Mini Kit (Qiagen) and on-column DNase digestion step was included to remove any residual DNA contamination according to the manufacturer’s instructions. For cDNA synthesis, the iScript cDNA Synthesis Kit (BIO-RAD) was used per user’s manual. The expression level of five selected transcripts was quantified in the kidney of infected brown trout and infected rainbow trout at different time points (6–12 wpe).

PCR primers specific for target genes were designed (Table 1) using primer design tool of NCBI Primer-BLAST (http://www.ncbi.nlm.nih.gov/tools/primer-blast/). PCR assays were optimized using gradient PCRs to determine the optimal annealing temperature and primer concentration. A CFX96 Touch Real-Time PCR detection system (BIO-RAD) was used to quantify gene expression levels in the kidney samples using iQ SYBR Green Supermix (BIO-RAD). A qRT-PCR in a final volume of 20 μl contained 2 μl of 1:10 fold diluted cDNA, 0.4 μM of each primer, 1× SYBR Green Supermix, and sterile distilled water. After 5 min of cDNA denaturation at 95 °C, 38 cycles were performed at 95 °C for 30 s, 55 °C for 30 s, and 72 °C for 30 s. A melting-point curve followed the cycling, starting from 55 °C and increasing by 0.5 °C every 10 s up to 95 °C, was used to detect any non-specific PCR products. Each qRT-PCR was performed in triplicate. Trout beta-actin was used as a reference gene for normalization (Rucker and El-Matbouli 2007). Standard curves were constructed for target genes and beta-actin gene with twofold serial dilutions of cDNA.

**Table 1:** Nucleotide sequence of quantitative real-time PCR primers used in this study

Primer name	Sequence (5′-3′)	Product size (bp)
TSC22D3 F	TGGCATTAACCTACCGCACT	156
TSC22D3 R	AATGCTTCTCGCCACGTTTG	
IgM H F	ACTGCTCCGACTTTGTTCCC	160
IgM H R	CCGCAGGGTACTGAACGAAA	
Rab-11b F	TGG CAG CAC GGT AGT TTG TT	89
Rab-11b R	CAC ATG ACG AGT CTC CAG GC	
PTMA-α F	GCCCCTGTAACCTCTCTCCT	108
PTMA-α R	TGTGTACACGGACATTGGGT	
Beta thymosin F	TCGAACGAGAGACGCAACTT	81
Beta thymosin R	GTCCAAACATCAACACGGGG	

### Statistical analysis

The relative expression of target gene was analyzed in the kidney of brown trout and rainbow trout using a linear mixed effect model for the difference between time points. Adjustment for multiple comparisons was performed using Sidaks’s procedure. The differences between groups (control and infected) at each single time point were analyzed using *t* tests for independent samples with Bonferroni α-correction. Correlations between relative expression levels of target genes were analyzed by calculating the Pearson product-moment correlation coefficient. For all statistical tests, a *p* value <0.05 was regarded as significant. All statistical analyses were conducted with SPSS v20 software.

## Results

### Identification of ESTs from the SSH library

**Fig. 3:**
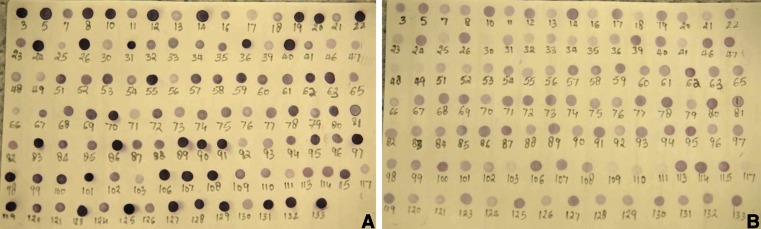
Dot blot hybridization of clones amplified by the adaptor-specific nested PCR. Denatured PCR products were dotted onto a positively charged nylon membranes, hybridized with DIG-labeled cDNA probes, and analyzed for differential screening. Positive dots of were identified as those that had different signal intensities as compared between subtracted cDNA libraries and control unsubtracted cDNA libraries. Dots with similar intensities were considered to be false positives. **a** Dots of the enriched subtracted cDNA library; **b** dots of the control unsubtracted cDNA library

Two hundred twenty clones were screened by adaptor-specific PCR: 108 from the forward-subtracted and 112 reverse-subtracted cDNA libraries. These amplified PCR products showed different size of inserts in the cDNA libraries. Sixty-one of 220 clones were identified as those had different signal intensities in dot blot differential screening (Fig. 3) and were selected for DNA sequencing: 39 “forward” clones and 22 “reverse” clones. Dots with similar signal intensities were considered to be false positives. NCBI BLAST searches showed that 9/61 clones (14.75 %) were similar to genes with immune function and defense mechanisms, ion transporter, endocytic pathway, cell proliferation, signal transduction, cell structure, and T cell activation (Tables 2 and 3). We examined five of these target genes using qRT-PCR. ESTs submitted to the GenBank dbEST database can be accessed under the following accession numbers: JZ713043 (prothymosin alpha), JZ713044 (GTP binding protein), JZ713045 (beta thymosin), JZ713046 (lymphocyte cytosolic protein 1), JZ713047 (TSC22 domain family protein 3), JZ713048 (Ras-related protein Rab-11B), JZ713049 (immunoglobulin mu), JZ713050 (FXYD domain containing ion transport regulator 5a), and JZ713051 (polyadenylate-binding protein 1). Twenty-one of 61 (34.42 %) and 16/61 clones (26.3 %), respectively, were most similar to the hemoglobin subunit alpha-4 and mitochondrial genes of trout. Eight of 61 clones (13.11 %) were identical with the trout cytochrome oxidase subunit 1. Three of 61 clones (4.91 %) were related to trout beta-actin; 2/61 (3.27 %) were trout coproporphyrinogen III oxidase; and 2/61 (3.27 %) were trout high choriolytic enzyme.

**Table 2:** cDNA sequences of transcripts revealed by suppressive subtractive hybridization of infected brown trout-tester and infected rainbow trout-driver (i.e., forward subtraction)

Genes	GenBank accession no.	Homolog species	Identity (%)	Functions	Dot blot analysis	
					BT	RT
TSC22 domain family protein 3	BT125136.1	*Salmo salar*	98	Anti-inflammatory and immunosuppressive	UP	DN
Immunoglobulin mu leavy chain	EF442498.1	*Oncorhynchus mykiss*	97	Humoral immune response	UN	UP
Ras-related protein Rab-11b	BT056918.1	*Salmo salar*	86	Endocytic pathway	DN	UP
FXYD domain containing ion transport regulator 5a	NM_001123723	*Salmo salar*	99	Ion channel activity	UP	DN
PolyA binding protein, cytoplasmic 1 b	XM_003968912.1	*Takifugu rubripes*	85	mRNA polyadenylation	UP	UN

*BT* brown trout, *RT* rainbow trout, *UR* upregulated, *DR* downregulated, *UN* unchanged

**Table 3:** cDNA sequences of transcripts revealed by suppressive subtractive hybridization of infected rainbow trout-tester and infected brown trout-driver (i.e., reverse subtraction)

Genes	GenBank accession no.	Homolog species	Identity (%)	Functions	Dot blot analysis	
					BT	RT
Prothymosin-α	BT150018.1	*Salmo salar*	99	Cell proliferation	UP	DN
GTP-binding protein	XM_005156728.1	*Danio rerio*	78	Signal transduction	DN	UP
Beta thymosin	NM_001124350.1	*Oncorhynchus mykiss*	99	Cell structure and motility	UN	UP
Lymphocyte cytosolic protein 1	AM600681.1	*Oncorhynchus mykiss*	84	T-cells activation and motility	DN	UP

*BT* brown trout, *RT* rainbow trout, *UR* upregulated, *DR* downregulated, *UN* unchanged

### Comparisons of transcript relative expression levels

The number of parasite stages and parasite quantity between both fish species are shown in Table 4; however, details are published in our previous study (Kumar et al. 2013). Dot blot screening data of nine ESTs are presented in Tables 2 and 3. The mean relative gene expression values of five target genes studied in both fish species are presented in Table 4 with parasite stages and parasite load at different time points. Table 4 shows differences in gene expression levels; however, TSC22 domain family protein 3 (TSC22D3) was found to be a main target gene that shows clear differences during presporogonic (6 wpe) and sporogonic stages of parasite in brown trout (8–12 wpe).

**Table 4:** Relative gene expression of five selected genes tested in this study with presporogonic stages, intra-luminal sporogonic stages, and relative parasite load

Genes		Different time points							
		6 wpe		8 wpe		10 wpe		12 wpe	
		BT	RT	BT	RT	BT	RT	BT	RT
	Presporogonic parasite stages	Low	Moderate	Low	High	Moderate	High	Low	Moderate
	Intra-luminal sporogonic stages	Very few	ND	High	ND	High	ND	Moderate	ND
	Relative parasite load	Low	High	High	High	High	High	High	Moderate
TSC22D3	R.E.	0.768	1.475	1.588	0.598	1.234	0.491	1.897	0.554
	*p* value	0.006		0.0001		0.0001		0.001	
IgM H	R.E.	3.984	5.742	12.815	7.201	2.404	9.757	1.840	2.994
	*p* value	0.004		0.003		0.002		0.050	
Rab-11b	R.E.	0.349	1.339	0.542	0.651	0.346	1.80	0.478	0.344
	*p* value	0.0001		0.366		0.0001		0.089	
PTMA-α	R.E.	1.116	1.096	1.354	0.615	0.745	0.832	1.803	0.623
	*p* value	0.869		0.001		0.191		0.0001	
Beta thymosin	R.E.	0.850	1.342	1.083	1.386	1.176	1.702	0.994	1.150
	*p* value	0.004		0.013		0.012		0.267	

Normalized mean values of relative gene expression (R.E.) in the kidney of infected brown trout (BT) and infected rainbow trout (RT) are presented with *p* values at different time points. The differences between groups at each single time point were analyzed using *t* tests for independent samples with Bonferroni α-correction

*ND* not detected

**Fig. 4:**
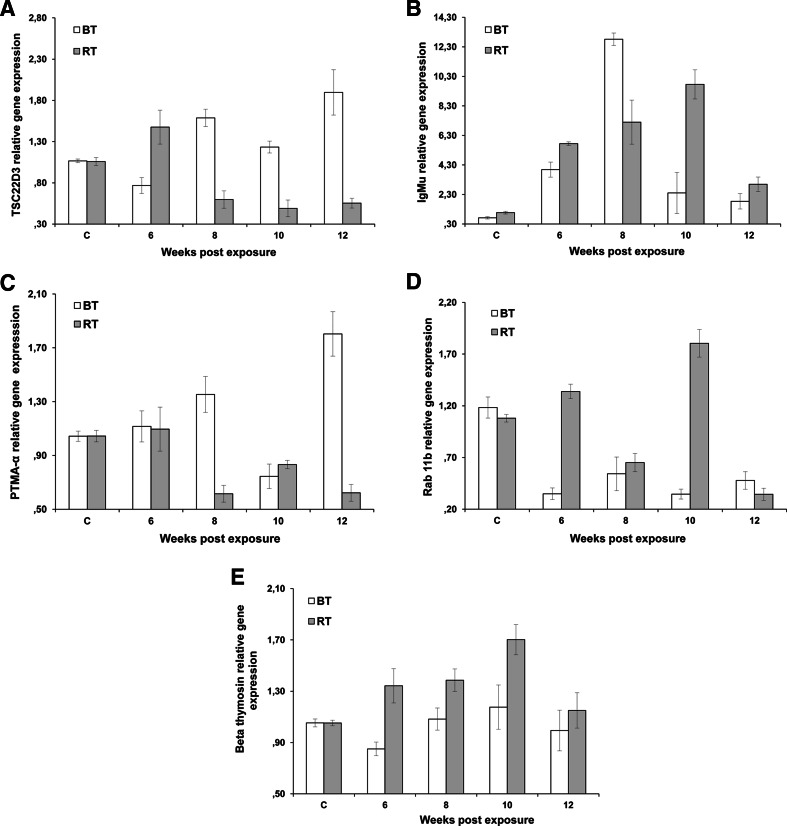
Quantitative real-time PCR of selected genes in brown trout *Salmo trutta* and rainbow trout *Oncorhynchus mykiss*. Comparison of relative gene expression profiles was performed in the kidney of brown trout (BT) and rainbow trout (RT) infected with *Tetracapsuloides bryosalmonae* at different time points. The qRT-PCR data were normalized to beta-actin expression. Relative gene expression changes were determined by calculating the mean expression values from the control and infected kidney samples. Each value represents the mean of triplicate independent biological samples, and *error bars* indicate standard deviation. **a** TSC22 domain family protein 3, **b** immunoglobulin mu, **c** Ras-related protein Rab-11b, **d** prothymin alpha, **e** beta thymosin

We found that immune function, defense mechanism, cell proliferation genes TSC22D3, immunoglobulin mu heavy chain (IgM H), and prothymin alpha (PTMA-α) were differentially expressed in the kidney of infected brown trout and rainbow trout at different time points. Expression of TSC22D3 was significantly downregulated at presporogonic stage (6 wpe) and upregulated (*p* < 0.018) in brown trout at sporogonic stages (8–12 wpe) compared to non-infected controls but significantly downregulated (*p* < 0.002) in rainbow trout at presporogonic stages (8–12 wpe) (Fig. 4a). Expression of IgM H was upregulated in brown trout and rainbow trout at all the time points compared to non-infected controls, while overall expression of IgM H was significantly higher (*p* < 0.05 or *p* < 0.002) in rainbow trout than brown trout at all time points except 8 wpe (Fig. 4b). Expression of PTMA-α was significantly upregulated (*p* < 0.018 or *p* < 0.001) in brown trout at 6, 8, and 12 wpe compared to non-infected controls but significantly downregulated (*p* < 0.001 or *p* < 0.002) in rainbow trout at 8–12 wpe (Fig. 4c). PTMA-α exhibited a significant positive correlation (*r* = 0.752; *p* < 0.0001) with TSC22D3 in brown trout.

We found that endocytic pathway and cell structure and motility genes Ras-related protein Rab-11b and beta thymosin were differentially expressed in the kidney of the two fish species: Ras-related protein Rab-11b was significantly upregulated (*p* < 0.005 or *p* < 0.001) in rainbow trout at 6 and 10 wpe compared to non-infected controls but significantly downregulated (*p* < 0.005) in brown trout at the all time points (Fig. 4d), which exhibited non-significant negative correlation with anti-inflammatory gene, TSC22D3 (*r* = −0.173; *p* = 0.359). Expression of beta thymosin was significantly upregulated (*p* < 0.021 or *p* < 0.001) in rainbow trout at 6, 8, and 10 wpe compared to non-infected controls, but its expression was neither significantly upregulated nor downregulated (*p* = 0.604 or *p* = 0.291) in brown trout at all time points except 6 wpe (Fig. 4e).

## Discussion

Previous studies have examined innate and adaptive immune genes such as inflammatory, antimicrobial, macrophage, antibody, and T helper cell marker genes in rainbow trout infected with European strain of *T. bryosalmonae* (Holland et al. 2003; Gorgoglione et al. 2013), but very little is known about gene expression in brown trout (Kumar et al. 2014). Herein, we report the first gene expression study of SSH identified genes in the kidney of brown trout and rainbow trout infected with the European strain of *T. bryosalmonae* at different time points. However, the gene expression studies in both fish species during infection with a North American strain of *T. bryosalmonae* is also required for comparison of their gene expression differences with a European strain of *T. bryosalmonae*.

In Europe, brown trout and rainbow trout respond differently to infection with *T. bryosalmonae*. Grabner and El-Matbouli (2008) and Kumar et al. (2013) demonstrated that rainbow trout infected with the European strain of *T. bryosalmonae* were unable to infect bryozoan *F. sultana* colonies and sporogonic stages of the parasite were not seen in the renal tubules of rainbow trout. Sporogonic stages of the parasite were detected in renal tubules of brown trout (Clifton-Hadley and Feist 1989; Holzer et al. 2006; Kumar et al. 2013). Recently, we reported remarkable differences in kidney swollen index, parasite numbers, and parasite quantity between brown trout and rainbow trout and also in transmission of parasite from infected fish to the bryozoan *F. sultana*. Such findings may confirm that both fish hosts express different gene responses against parasite infection (Kumar et al. 2013). In the present study, we observed significant transcriptional upregulation and downregulation of anti-inflammatory, humoral immune and endocytic pathway responses, cell proliferation, and cell growth processes in infected brown and rainbow trout, which may affect parasite sporogonic stages in the host.

Using differential screening, we detected 61 clones of which nine showed differential expression levels between infected brown trout and infected rainbow trout. Differential screening revealed a relatively high background of false-positive clones in both trials as confirmed by NCBI BLAST searches, which suggested that both transcriptomes were similar, and that only low numbers of genes were expressed differently during parasite development between both infected fish species. Our NCBI BLAST searches of the expressed sequence tags showed similarities with known fish genes to the order of 78–99 % at the nucleotide level. The nine differentially expressed genes that we identified have functions such as immune response and defense mechanisms, signal transduction activity, cell proliferation and apoptosis, cell structure and mobility, membrane protein transporter, and mRNA metabolism. Gene expression of transcripts was examined and compared in the kidney of brown trout at both presporogonic and sporogonic stages and rainbow trout only at presporogonic stages of parasite. We found variation in gene expression of transcripts in both fish species or within same fish species at different time points (Table 4). This may be from the fish host species differences, from the ability of each cell type to respond to different transcription factors, from the different epigenetic state of each cell type, or from immune cell regulatory networks and cell signalling pathways that affect gene expression of both fish species in response to presporogonic or sporogonic stages of parasite development.

One of the nine genes that we identified, a TSC22 domain family protein 3, is a regulatory protein that plays a role in the anti-inflammatory and immunosuppressive effects of glucocorticoids and interleukins, and in the apoptotic process (Vogel et al. 1996). A transcriptome study of Atlantic salmon infected with *Piscirickettsia salmonis* showed a decrease in kidney expression level of TSC22D3 48 h post-infection (Tacchi et al. 2011). Our qRT-PCR data showed that TSC22D3 was downregulated in the kidneys of rainbow trout at presporogonic stages and upregulated in the kidney of brown trout at sporogonic stages of parasite. This suggests that upregulation of TSC22D3 plays an important anti-inflammatory role in kidney of brown trout during sporogonic stages (8–12 wpe), whereas its downregulation supports an inflammatory response in the kidney of rainbow trout during presporogonic stages (8–12 wpe) except 6 wpe. These observations are in concordance with PKD signs in our previous study, where we found that the kidneys of rainbow trout but not brown trout were intensely swollen at 8–12 wpe (Kumar et al. 2013). In the study of Gorgoglione et al. (2013), anti-inflammatory cytokines genes such as interleukin (IL)-6 and IL-11 were highly upregulated in the kidney of rainbow trout, where fish were exposed to natural parasite infected water in early April and sampled in late July, i.e., approximately 14 wpe. It may be possible that TSC22D3 upregulates at 14 wpe in rainbow trout and downregulates at 17 wpe because in our previous study, it has been shown that kidney swelling of rainbow trout was moderate at 14 wpe but normal at 17 wpe as well as parasite stages, and parasite load was confirmed at 14 wpe but not at 17 wpe (Kumar et al. 2013).

Immunoglobulin (as IgM H) was upregulated in infected kidneys of brown trout and rainbow trout. Immunoglobulin is used by the immune system to identify and neutralize microorganisms. In Europe, mainly in the UK, the expression of innate and adaptive immune genes was studied in rainbow trout following a natural exposure to *T. bryosalmonae*. Expression of secretory forms of IgM and IgT are markedly upregulated in the kidney of rainbow trout, and their expression levels correlate with parasite prevalence and kidney swelling (Gorgoglione et al. 2013). However, other myxozoans such as *E. leei* elicits downregulation of IgM in the kidney of gilthead sea bream (Estensoro et al. 2012). We found that expression of IgM H was significantly higher in rainbow trout than brown trout, which implied higher activation of B cells in rainbow trout, to produce massive amount of antibodies in response to parasite infection. This is likely to have a broad spectrum of activity to suppress sporogenesis of the parasite in the kidney lumen of rainbow trout. Furthermore, cytokines, IL-6, IL-10, and IL-11 stimulate humoral immune responses during parasite infection (Gorgoglione et al. 2013) that could cause differences in expression level of immunoglobulins and humoral immune responses in both fish species during PKD pathogenesis.

In the present study, expression of Ras-related protein (Rab-11b) was generally upregulated in rainbow trout but downregulated in brown trout. Rab-11b is a key regulator of intracellular membrane trafficking and endocytic recycling (Bos 1997). Rab family proteins have been involved in immune defense responses of red drum (*Sciaenops ocellatus*) against microbial infections and affected the entry levels of intracellular pathogens in hosts (Hu et al. 2011). Rab1 is upregulated in the kidney of red drum in response to intracellular bacteria *Edwardsiella tarda* during 4- to 24-h time periods of infection (Hu et al. 2011), and Rab-1A, Rab-6A, and Rab-10 proteins have been upregulated persistently in the liver of gilthead sea bream profile after confinement exposures (Calduch-Giner et al. 2010). We found that expression of Rab-11b was upregulated in rainbow trout but downregulated in brown trout at all time points. Upregulation of intracellular membrane trafficking and endocytic recycling in rainbow trout may play an important role in immune defense mechanisms including the renal membrane remodeling and fusion, entry and intracellular transport processes, and regulation of several related pathways. We can assume that downregulation of intracellular membrane trafficking and endocytic recycling activities in brown trout may support the development of sporogonic stages of *T. bryosalmonae*. However, the role of Rab-11b protein in the development of parasite in fish host is unclear and needs to be elucidated.

GTP binding protein was downregulated in brown trout but upregulated in rainbow trout infected with *T. bryosalmonae* using dot blot analysis. GTP-binding proteins, also known as G proteins, are a family of proteins belonging to the larger group of enzymes called GTPases, which are involved in transmitting signals from a variety of different external stimuli to the inside of a cell. G proteins are activated by G protein-coupled receptors that regulate metabolic enzymes, ion channels, transporters, and other parts of the cell machinery (Krauss 2008). GTPases proteins such as Ras, Rap, and Rho are involved in regulating the function of the immune system such as T cells, B cells, and dendritic cells (Scheele et al. 2007). Our findings suggest that the expression of genes involved in signal transduction regulate immune responses, cell migration, and apoptosis, which may suppress parasite stages in the kidney of rainbow trout. However, downregulation of these genes may support the development of sporogonic stages in the kidney of brown trout. Nevertheless, functional experiments are needed to verify the precise roles of GTP-binding proteins in the development of parasite sporogonic stages in fish.

We found significant upregulation of PTMA-α in the kidney of brown trout but not in rainbow trout. PTMA-α is an abundant small acidic nuclear protein widely distributed in mammalian cells and tissues. It is associated with cell proliferation, protection against apoptosis, and chromatin remodeling activity (Eschenfeldt and Berger 1986). PTMA-α induces T cell maturation and differentiation in response to antigens (Baxevanis et al. 1990). Moreover, PTMA-α upregulates MHC class II gene expression in various cell types and may mediate immune function by blocking the effect of PTMA-α, which confers resistance to certain opportunistic infections (Baxevanis et al. 1992). Overexpression of PTMA-α accelerates cell proliferation, and inhibition of PTMA-α synthesis prevents cell division and induces apoptosis (Jiang et al. 2003). Our finding of upregulation of PTMA-α in brown trout suggests that cell proliferation and cell growth processes in that host do not impede parasite sporogony, which exhibited a significant positive correlation with anti-inflammatory gene, TSC22D3. We previously demonstrated that infection with *T. bryosalmonae* influences the growth of rainbow trout but not brown trout (Kumar et al. 2013).

We found several important proteins involved in cell structure and motility, e.g., beta thymosins, which is a family of highly conserved small proteins with multiple functions (Huff et al. 2001). Beta thymosins play a crucial role in many cellular functions such as tissue regeneration, cell shape change, cell motility, stimulation of pro-inflammatory cytokine secretion, and activation of the c-Jun N-terminal kinase signaling pathway (Zhang et al. 2008). Upregulation of beta thymosin has been observed in hemocytes of black tiger shrimp (*Penaeus monodon*) in response to white spot and yellow head viruses, and *Vibrio harveyi* (Pongsomboon et al. 2011). We found beta thymosin upregulation in rainbow trout but not in brown trout, suggesting an important role in renal regeneration and supports the pro-inflammatory cytokine environment in rainbow trout. This is in agreement with the study of Gorgoglione et al. (2013) that expression of pro-inflammatory cytokine genes such as IL-1β1 and TNF-α2 were upregulated significantly in infected rainbow trout at kidney swelling grade 1 during PKD pathogenesis.

Lymphocyte cytosolic protein 1 (LCP1) was differentially expressed in the two fish species. LCP1, also known as L-plastin/LPL, is a family of actin-binding proteins expressed only in hematopoietic cells. It regulates cytoskeletal re-arrangements and NADPH oxidase, which are essential for the antimicrobial respiratory burst functions (Chen et al. 2003). LCP1 has been shown to have a role in T cell activation and motility. T cells are a type of lymphocyte, a subset which includes different types of T cells that play a central role in cell-mediated immunity (Morley 2013). LCP1 has been upregulated in zebrafish (*Danio rerio*) in response to *Mycobacterium marinum* infection (Meijer et al. 2005). Interestingly, expression of plastin-2/L-plastin was downregulated in the head kidney of gilthead sea bream infected with *E. leei* (Davey et al. 2011). We found that LCP1 was downregulated in brown trout using dot blot analysis, which may support the development of sporogonic stages of the parasite, whereas it was upregulated in rainbow trout, which may play a central role in the activation of T cells/cell-mediated immune responses against *T. bryosalmonae* infection in that host.

We found a transport protein was differentially amplified in the fish. This is a small membrane protein involved in the movement of ions across a plasma membrane, e.g., FXYD domain-containing ion transport regulator 5a, which functions as ion channel and tissue-specific regulatory subunits of the Na^+^-K^+^-ATPase (Geering 2006). In the renal tubules, the Na^+^ gradient is generated by Na^+^-K^+^-ATPase, which drives various ion and nutrient transporters that accomplish ion, osmoregulation, and nutrient uptake (Feraille and Doucet 2001). We found FXYD 5a upregulation in brown trout and downregulation in rainbow trout infected with *T. bryosalmonae* using dot blot analysis, suggesting that downregulation of ion channel activities/transport proteins reduces cell membrane Na^+^-K^+^-ATPase activity in the kidney of rainbow trout. Its upregulation in brown trout probably enhances the renal tubular epithelial Na^+^-K^+^-ATPase activity that maintains cell membrane, cell structure, and the cellular environment for the development of parasite sporogonic stages in that host.

We found polyadenylate-binding protein cytoplasmic 1 (PABPC1) to be differentially expressed in brown trout and rainbow trout. PABPC1 is a cytoplasmic-nuclear shuttling protein, which is important for RNA polyadenylation, translation initiation, and mRNA stability (Mangus et al. 2003). mRNA translation is the most energy-demanding process in the cell and strongly correlates with cellular metabolic activities (Buttgereit and Brand 1995). PolyA binding protein is upregulated in hemocytes of Japanese tiger shrimp in response to microbial infections (He et al. 2004). Our data on PABPC1 expression in fish host shows that mRNA translation is affected during the infection of *T. bryosalmonae*, thus impacting cellular metabolic activities in the kidney tissue of the host. Davey et al. (2011) report that host translation proteins such as 28S, 40S, and 60S ribosomal proteins were either upregulated or downregulated in the head kidney of gilthead sea bream in response to *E. leei* infection.

In conclusions, we identified nine transcripts that were differentially expressed in the kidneys of infected brown trout and rainbow trout. Gene expression was either upregulated or downregulated during the course of *T. bryosalmonae* development. These results suggest that differential expression of host anti-inflammatory, humoral immune and endocytic pathway responses, cell proliferation, and cell growth processes do not inhibit the development of intra-luminal sporogonic stages of the European strain of *T. bryosalmonae* in brown trout but may suppress it in rainbow trout. The present study provides fundamental information for understanding trout kidney tissue response, candidate genes, and their modulation during the parasite infection. These genes may also play a role in host defense against other malacosporean species. Further study is required to investigate the role of some important candidate genes, their effect, and their associated pathways in the development or inhibition of sporogonic stages of *T. bryosalmonae* in the kidney of fish host.
